# Association of household income and education with eating behaviors in Japanese adults: a cross-sectional study

**DOI:** 10.1186/s12889-016-2748-z

**Published:** 2016-01-22

**Authors:** Saki Nakamura, Takayo Inayama, Kikuko Hata, Munehiro Matsushita, Masaki Takahashi, Kazuhiro Harada, Takashi Arao

**Affiliations:** 1 Graduate School of Human Health Sciences, Tokyo Metropolitan University, Hachioji, Tokyo Japan; 2 School of Sports Sciences, Waseda University, Tokorozawa, Saitama Japan; 3 Faculty of Science and Engineering, Waseda University, Shinjyuku, Tokyo Japan; 4 Section of Motor Function Activation, National Center for Geriatrics and Gerontology, Obu, Aichi Japan

**Keywords:** Japan, Eating behaviors, Socioeconomic status, Household income, Education

## Abstract

**Background:**

Socioeconomic inequalities as social determinants of health are important issues in public health and health promotion. However, the association between socioeconomic status and eating behaviors has been investigated poorly in Japanese adults. To fill this gap, the present study examines the association of eating behaviors with household income and education.

**Methods:**

The sample comprised 3,137 Japanese adults (1,580 men and 1,557 women) aged 30 to 59 years who responded to an Internet-based cross-sectional survey in 2014. Data on the following eating behaviors were collected via self-report: “taking care of one’s diet for health,” “eating vegetables,” “frequency of eating breakfast,” “frequency of family breakfasts,” “frequency of family dinners,” “using the information on nutrition labels,” and “conversations with family or friends during meals.” Self-reported data on socioeconomic status (household income and education) and demographic variables (gender, age, district of residence, marital status, residence status, and employment status) were also collected. The associations between eating behaviors and household income or education were tested using binomial logistic regression analysis with eating behaviors as dependent variables and household income and education as independent variables. A trend P -value was calculated for three categories of household income (less than 3,000,000 JPY, 3,000,000–7,000,000 JPY, and over 7,000,000 JPY) and education (junior high/high school, 2-year college, and 4-year college/graduate school).

**Results:**

Higher household income and education were significantly associated with higher rates of eating vegetables, using the information on nutrition labels, and conversation with family or friends during meals in Japanese men and women. Higher household incomes were significantly associated with lower rates of frequency of family breakfasts in Japanese men and lower rates of frequency of family dinners in Japanese men and women.

**Conclusions:**

Higher socioeconomic status as indicated by household income or education was associated with eating more vegetables and conversation with family or friends during meals in Japanese men and women. Socioeconomic status should be considered in health promotion and diet improvement.

## Background

Health disparities are important issues in public health and health promotion. Socioeconomic disparities in health have been widely reported in many Western countries. In several previous studies, individual socioeconomic status (SES), as measured by household income, education, or occupation, has been shown to be closely related to lifestyle, mortality, and morbidity [1, 2]. In Japan, reduction of health disparities caused by differences in SES is considered important for public health and health promotion [3, 4]. Until recently, few major socioeconomic disparities have been reported among the Japanese population [5]. Several recent reports, however, have shown that a lower SES is generally associated with higher likelihood of behaviors with a health risk [6, 7] as well as higher mortality and morbidity [8–11]. A review of the research on health differences will therefore support measures to reduce health disparities in Japan.

The World Health Organization (WHO) [12] identified the main intermediary determinants of health, usually behavioral or social factors, as nutritional and dietary behaviors, physical activity, tobacco consumption, and alcohol consumption. These factors differ by SES; dietary habits, for example, are an important factor in disease prevention and health promotion among those with low SES [13–15]. Fewer of those with a household income of less than 2,000,000 JPY have an adequate vegetable intake than those whose household income is more than 6,000,000 JPY [16]. Murakami [17] reported that lower education levels and working outside the home were associated with an unfavorable dietary intake pattern in a group of pregnant Japanese women. Fukuda [18] reported that lower household expenditure was associated with unhealthy and unbalanced nutrient intake in Japanese adults. These differences in nutritional intake were also predicted by sex, age, and marital status. Health status and nutrition are affected by food intake, which is in turn affected by eating behaviors. The relationship between SES and dietary behavior has a number of known mediators and moderators [12], but the relationships between eating behaviors and SES in Japan are largely unexplored. As a mechanism to transform dietary behavior, we applied the Healthy Japan 21 policy framework of goal-setting for eating habits [12, 19].

Dietary behavior is affected by knowledge, skills, attitudes, food preparation factors and environment [19]. Nutrition education aims to transform eating behavior. It is necessary to measure socioeconomic factors, such as household income or education, to promote desirable action and transformation [4]. By examining the association between healthy eating behaviors and SES, we can set SES-specific action targets to support changes in eating patterns. There are, however, very few studies on the association between household income or education and eating behavior [16], although it has been reported that the frequency of eating breakfast differs by sex and household income [16]. To obtain basic data as a foundation for research on health disparities, it is first necessary to establish whether healthy eating behaviors differ by sex, as in previous studies [17, 18], and to examine the association between SES (household income and education) and healthy eating behavior. If we can establish the relationship between dietary behavior and SES, this will inform the development of future SES-based interventions.

We hypothesized that people with higher SES would have healthier eating habits, which would in turn influence their general health, because higher SES permits adequate intake of healthy food and therefore good health. We used seven items chosen from the Japan National Health and Nutrition Survey (NHNS) [16] and previous studies [20–30]. Other projects include a child poverty program [31] and the Japan Gerontological Evaluation Study (JAGES) [32]. As adults are responsible for raising the next generation, we decided that targeting adults would have a longer term effect on reducing health disparities. We therefore examined differences in eating behaviors among adults with different SES. Associations between SES and eating behaviors in Japan may differ from those found in other countries because of variations in socioeconomic conditions. We therefore examined the association between SES and healthy eating behaviors in a sample of Japanese adults, to provide support for interventions to improve dietary habits in Japan.

## Methods

### Participants

An Internet-based cross-sectional survey was conducted in February 2014 by a Japanese online research service company that holds data on approximately 160,300 adult registrants, including their sociodemographic attributes. This allows the company to target particular demographic groups as necessary. We aimed to have a sample of approximately 3,000 adults aged 30 to 59 years, with 500 men and 500 women from three age groups (30–39, 40–49, and 50–59 years). We targeted adults aged 30 to 59 because we felt that both promotion of healthy eating and reduction in health disparities were particularly important in this group. The set sample size and attributes were stratified by distribution of the Japanese average age, the 2013 Population Census of Japan for sex [33], and the 2012 Comprehensive Survey of Living Conditions in Japan for household income [34]. In total, 8,284 adults were randomly selected from the database and received an e-mail inviting them to participate in our survey. The invitation e-mail contained a URL directing potential respondents to a protected area of the website where the questionnaire was located. They could then log on using their ID and password. The research service company offered rewards points valued at 100 JPY (one USD was equivalent to approximately 102 JPY in February, 2014). Of those invited, 3,269 answered the survey questions online (a response rate of 39.5 %). Respondents who completed the questionnaire and clicked on the Send button at the end of the online informed consent form were considered to have agreed to participate in the survey. The study received prior approval from the Ethics Review Committee on Research with Human Subjects of Waseda University, Japan.

### Socioeconomic status and sociodemographic variables

Household income and educational level were used to measure SES. Educational level was grouped into three categories: junior high/high school, 2-year college, and 4-year college/graduate school. We were unable to accurately ascertain individual-level equivalent incomes because the choices were for levels of household income: less than 3,000,000 JPY, 3,000,000–5,000,000 JPY, 5,000,000–7,000,000 JPY, 7,000,000–10,000,000 JPY, 10,000,000–15,000,000 JPY, and over 15,000,000 JPY. We therefore analyzed income at category level. Household income was classified into three equally distributed categories: less than 3,000,000 JPY, 3,000,000–7,000,000 JPY, and over 7,000,000 JPY.

### Eating behavior

Seven items were used to measure eating behavior: taking care of one’s diet for health, eating vegetables [20, 21], frequency of eating breakfast [22, 23], frequency of family breakfasts and dinners [24–26], using the information on nutrition labels [27, 28], and conversations with family or friends during meals [29, 30]. These dietary behaviors were in line with the aims of programs like Healthy Japan 21. Questions about eating behaviors were preceded by the phrase, “The following questions are about your normal meals”. Respondents were asked, “Do you normally take an interest in nutrition and healthy meals?” There were six response choices: (1) Very often; (2) Often; (3) Sometimes; (4) Rarely; (5) Almost never; and (6) Never. Participants who answered (1) to (3) for this question were defined as those taking care of one’s diet for health. Respondents were asked, “Do you eat adequate amounts of vegetables (5 small dishes/day, or about 350 g)?” Responses included the following four choices: (1) Very often; (2) Often; (3) Not much; and (4) Never. People who answered (1) or (2) to this question were defined as those who eat sufficient amounts of vegetables.

The next questions were preceded by the phrase, “The following questions are about your normal lifestyle”. Respondents were asked, “How often do you usually eat breakfast?”, with five possible responses: (1) Every day; (2) 4 or 5 days/week; (3) 2 or 3 days/week; (4) 1 day/week; and (5) Never. Participants who answered (1) to this question were defined as those who eat breakfast regularly. Respondents were asked, “How often each week do you usually eat breakfast with all the members of your family?” and “How often each week do you usually eat dinner with all the members of your family?” The following five response choices were provided: (1) Every day; (2) 4 or 5 days/week; (3) 2 or 3 days/week; (4) 1 day/week; and (5) Never. People who answered (1) were defined as those who regularly eat meals with their families each question.

The final questions were preceded by, “The following questions are about your normal eating habits”. Respondents were asked, “Do you use the information on nutrition labels or calorie information on store displays and menus?” Possible responses were: (1) Very often; (2) Often; (3) Not much; and (4) Never. Participants who answered (1) or (2) were defined as those who consult nutrition information. Respondents were asked, “Do you talk to your family and friends during meals about the meal or nutrition?” with four response choices: (1) Very often; (2) Often; (3) Not much; and (4) Never. People who answered (1) or (2) to this question were defined as having positive conversations during meals.

### Health risk behaviors

The study asked about two health-risk behaviors: current smoking and alcohol consumption. Smoking habits were surveyed with the question, “How many cigarettes or cigars per day do you smoke?” Responses were: (1) I have never smoked; (2) I stopped smoking more than 1 year ago; (3) I stopped smoking less than 1 year ago; (4) I smoke 1–20 cigarettes or cigars per day (5) I smoke 21–40 cigarettes or cigars per day; and (6) I smoke more than 41 cigarettes or cigars per day. We categorized participants who responded (4) to (6) as current smokers. Alcohol consumption was surveyed by asking, “How many days per week do you consume alcohol?” Response choices were as follows: (1) Every day; (2) 5 or 6 days/week; (3) 3 or 4 days/week; (4) 1 or 2 days/week; (5) 1 to 3 days/month; (6) I stopped consuming alcohol more than 1 year ago; and (7) I hardly drink alcohol at all. We categorized responses (1) to (5) as current alcohol consumption.

### Demographic variables

Demographic variables included sex, age, marital status, residence status, and employment status. Age was classified as 30–39, 40–49, and 50–59 years. Marital status was categorized as currently married or currently unmarried. Residence status was categorized as living with others or living alone. Employment status was categorized as employed or not employed.

### Data analysis

Data were analyzed for the 3,137 adults who provided complete information for the study variables. Respondents who did not provide education status (Other/Unknown, *n* = 52) or employment status (Other/Unknown, *n* = 80) were not included in the analysis because these were important variables in this study. We considered responses of Other/Unknown to be missing values rather than lost data. Interpretation of the results would have been difficult if Other/Unknown responses were combined with the other choices, not least because there were very few such responses. We therefore excluded these data from the analysis.

Statistical analysis was performed overall and separately by sex. The chi-squared test was used to compare various characteristics and eating behaviors between men and women. Associations between SES and healthy eating behaviors were examined using forced-entry adjusted logistic regression analysis. Unadjusted odds ratios (OR), adjusted odds ratios (AOR), and 95 % confidence intervals (CI) were calculated for each variable. Associations between eating behaviors and household income or education were determined using binomial logistic regression analysis, with eating behaviors as the dependent variable and household income and education as independent variables. Household income as an independent variable was adjusted for age group; marital, residence and employment status; and education. Education as an independent variable was adjusted for age group; marital, residence and employment status; and household income. Previous studies have found that differences in eating behaviors were predicted by sex, age, marital status, and residence status [6, 17, 18], and have also adjusted for sex, age, and residence status [16]. We also adjusted for employment status for two reasons, first, because nutrient intake and being employed have previously been shown to be related in women [17], and second because being employed influences household income and its relationships to dietary behavior. We did not adjust for alcohol consumption and smoking because the associations did not change when adjusted. A trend p-value was calculated for the three categories of household income and education, and p values of < 0.05 were considered significant. IBM SPSS Statistical Package for Windows Version 21.0 (IBM Japan Inc., Tokyo, Japan) was used for all statistical analyses.

## Results

Table 1 shows demographic characteristics of the 3,137 respondents. Overall mean age was 44.1 (SD = 8.1) years and was approximately the same for men and women. Substantial proportions were current smokers and consumers of alcohol (28.0 % and 72.3 % of men, 13.5 % and 50.2 % of women).

**Table 1:** Characteristics of respondents and health behaviors

Variables	Group	Total		Men		Women		P value
		*n* = 3137		*n* = 1580		*n* = 1557		
		n	%	n	%	n	%	
Basic characteristics of study subjects								
Gender	Men	1580	50.4					
	Women	1557	49.6					
Age	30–39	1072	34.2	540	34.2	532	34.2	0.926
	40–49	1117	35.6	567	35.9	550	35.3	
	50–59	948	30.2	473	29.9	475	30.5	
Marital status	Not married^b^	1249	39.8	736	46.6	513	32.9	<0.001***
	Married	1888	60.2	844	53.4	1044	67.1	
Residence status	Not living together	555	17.7	365	23.1	190	12.2	<0.001***
	Living together	2582	82.3	1215	76.9	1367	87.8	
Employment status^a^	Employed	2308	73.6	1405	88.9	903	58.0	<0.001***
	Not employed	829	26.4	175	11.1	654	42.0	
Household income	<3,000,000 JPY	984	31.4	501	31.7	483	31.0	0.862
	3,000,000–7,000,000 JPY	1293	41.2	644	40.8	649	41.7	
	>7,000,000 JPY	860	27.4	435	27.5	425	27.3	
Educational status	Junior high/high school	830	26.5	412	26.1	418	26.8	<0.001***
	Two-year college	866	27.6	257	16.3	609	39.1	
	4-year college/graduate school	1441	45.9	911	57.7	530	34.0	
Health behaviors								
Smoking behavior	Nonsmokers	2485	79.2	1138	72.0	1347	86.5	<0.001***
	Current smokers	652	20.8	442	28.0	210	13.5	
Drinking habits	Nondaily	1213	38.7	437	27.7	776	49.8	<0.001***
	Drinking	1924	61.3	1143	72.3	781	50.2	

*P < 0.05; **P < 0.01; ***P < 0.001

^a^Not married: single, separated, or divorced

^b^Chi-square test

The prevalence of each eating behavior is shown in Table 2. Nearly 75 % of participants reported a positive intention to take care of their diet for the sake of their health. The frequency of eating breakfast was higher frequency. Most participants reported a lower frequency of eating vegetables, family breakfasts, family dinners, using nutrition information, and positive conversations with family or friends during meals. The eating behaviors of men and women differed significantly.

**Table 2:** Prevalence of eating behaviors in Japanese adults

Variables	Group^a^	Total		Men		Women		
		*n* = 3137		*n* = 1580		*n* = 1557		P value
		n	%	n	%	n	%	
About eating behavior								
Taking care of one’s diet for health	Very often, often, sometimes	2306	73.5	1066	67.5	1240	79.6	<0.001***
Taking care of one’s diet for health (Do you normally concern yourself with nutrition and meals for your own health?)	Rarely, almost never, never	831	26.5	514	32.5	317	20.4	
Behavior about the meal								
Eating vegetables	Very often, often	1453	46.3	631	39.9	822	52.8	<0.001***
Eating vegetables (Do you eat ample amounts of vegetables [5 small dishes/day, or about 350 g]?)	Not much, never	1684	53.7	949	60.1	735	47.2	
Frequency of eating breakfast	Every day	2132	68.0	972	61.5	1160	74.5	<0.001***
Frequency of eating breakfast (How often do you usually eat breakfast?)	4 or 5 days/week, 2 or 3 days/week, 1 day/week, never	1005	32.0	608	38.5	397	25.5	
Frequency of family breakfasts	Every day	906	28.9	351	22.2	555	35.6	<0.001***
Frequency of family breakfasts (How often do you usually eat breakfast with all the members of your family each week?)	4 or 5 days/week, 2 or 3 days/week, 1 day/week, never	2231	71.1	1229	77.8	1002	64.4	
Frequency of family dinners	Every day	1374	43.8	530	33.5	844	54.2	<0.001***
Frequency of family dinners (How often do you usually eat dinner with all the members of your family each week?	4 or 5 days/week, 2 or 3 days/week, 1 day/week, never	1763	56.2	1050	66.5	713	45.8	
Meal information exchange/utilization								
Using the information on nutrition labels	Very often, often	1453	46.3	600	38.0	853	54.8	<0.001***
Using the information on nutrition labels (Do you use the information on nutrition labels or calorie information on store displays and menus?	Not much, never	1684	53.7	980	62.0	704	45.2	
Conversations with family or friends during meals	Very often, often	1326	42.3	532	33.7	794	51.0	<0.001***
Conversations with family or friends during meals (Do you talk to your family and friends about meals, cooking, or nutrition?)	Not much, never	1811	57.7	1048	66.3	763	49.0	

*P < 0.05; **P < 0.01; ***P < 0.001

^a^Chi-square test

Table 3 shows the results of logistic regression analysis of the association between household income and eating behaviors. In the unadjusted analysis, taking care of one’s diet for health, eating vegetables, frequency of eating breakfast, frequency of family breakfasts, using information on nutrition labels, and positive conversations with family or friends during meals were positively associated with higher household income. After adjusting for all variables, frequency of family breakfast and dinners were both negatively associated with higher household income. Frequency of eating breakfast was not associated with household income. For all income categories, all domains except frequency of eating breakfast showed significant trends.

**Table 3:** Association of household income with eating behaviors in Japanese: Unadjusted odds ratio (OR) and adjusted odds ratio (OR) and 95 % confidence interval (95 % CI)

Dependent variables^||^	Independent variables^‡^								
	Household income								
		Unadjusted^§^			Trend p-value^†^	Adjusted^§^			Trend p-value^†^
	Group	OR	95 % CI	p-value		OR	95 % CI	p-value	
Taking care of one’s diet for health^a^	<3,000,000 JPY	1.00	(ref)		<0.001***	1.00	(ref)		<0.001***
	3,000,000 JPY–7,000,000 JPY	1.53	1.27–1.83	<0.001***		1.43	1.17–1.75	0.001**	
	>7,000,000 JPY	2.28	1.84–2.83	<0.001***		1.98	1.55–2.54	<0.001***	
Eating vegetables^b^	<3,000,000 JPY	1.00	(ref)		<0.001***	1.00	(ref)		<0.001***
	3,000,000 JPY–7,000,000 JPY	1.76	1.48–2.09	<0.001***		1.51	1.25–1.83	<0.001***	
	>7,000,000 JPY	2.32	1.92–2.80	<0.001***		1.79	1.44–2.22	<0.001***	
Frequency of eating breakfast^c^	<3,000,000 JPY	1.00	(ref)		0.002**	1.00	(ref)		0.498
	3,000,000 JPY–7,000,000 JPY	1.43	1.20–1.70	<0.001***		1.10	0.90–1.34	0.365	
	>7,000,000 JPY	1.35	1.11–1.64	0.003**		0.94	0.75–1.18	0.592	
Frequency of family breakfasts^c^	<3,000,000 JPY	1.00	(ref)		0.001**	1.00	(ref)		0.019*
	3,000,000 JPY–7,000,000 JPY	1.47	1.22–1.78	<0.001***		0.87	0.70–1.08	0.192	
	>7,000,000 JPY	1.43	1.16–1.75	0.001**		0.73	0.58–0.94	0.013*	
Frequency of family dinners^c^	<3,000,000 JPY	1.00	(ref)		0.443	1.00	(ref)		<0.001***
	3,000,000 JPY–7,000,000 JPY	1.38	1.17–1.64	<0.001***		0.76	0.62–0.94	0.010*	
	>7,000,000 JPY	1.06	0.88–1.28	0.551		0.51	0.40–0.65	<0.001***	
Using the information on nutrition labels^b^	<3,000,000 JPY	1.00	(ref)		<0.001***	1.00	(ref)		<0.001***
	3,000,000 JPY–7,000,000 JPY	1.29	1.09–1.53	0.003**		1.24	1.03–1.50	0.024*	
	>7,000,000 JPY	1.80	1.50–2.17	<0.001***		1.63	1.31–2.02	<0.001***	
Conversations with family or friends during meals^b^	<3,000,000 JPY	1.00	(ref)		<0.001***	1.00	(ref)		<0.001***
	3,000,000 JPY–7,000,000 JPY	1.46	1.23–1.73	<0.001***		1.10	0.91–1.33	0.342	
	>7,000,000 JPY	2.20	1.82–2.65	<0.001***		1.47	1.18–1.83	0.001**	

*P < 0.05; **P < 0.01; ***P < 0.001

^†^Trend test

^‡^The independent variable of household income was adjusted for gender, age classification, marital status, residence status, employment status and education

^§^OR = odds ratio; CI = confidence interval; ref = referent group

^||^As the dependent variable of dietary behaviors, seven items were confirmed the distribution of the answer and categorized in positive answer = 1, negative answer = 0

^a^Responses were given in six categories: (1) very often; (2) often; (3) sometimes; (4) rarely; (5) almost never; and (6) never. People who answered (1) to (3) to the question were defined as positive answer, (4) to (6) to the question were defined as negative answer

^b^Responses were in the following four categories: (1) very often; (2) often; (3) not much; and (4) never. People who answered (1) or (2) to the question were defined as positive answer, (3) or (4) to the question were defined as negative answer

^c^Responses were rated in the following five categories: (1) every day; (2) 4 or 5 days/week; (3) 2 or 3 days/week; (4) 1 day/week; and (5) never. People who answered (1) to the question were defined as positive answer, (2) or (5) to the question were defined as negative answer

In the unadjusted analysis (Table 4), taking care of one’s diet for health, eating vegetables, using nutrition information, and positive conversations during meals were positively associated with completing 4-year college or graduate school. Frequency of family breakfasts and dinners were negatively associated with completing 4-year college or graduate school. After adjusting for all variables, frequency of family dinners was not associated with education. For all education levels, all domains except frequency of eating breakfast and frequency of family dinners showed significant trends.

**Table 4:** Association of education with eating behaviors in Japanese: Unadjusted odds ratio (OR) and adjusted odds ratio (OR) and 95 % confidence interval (95 % CI)

Dependent variables^||^	Independent variables^‡^								
	Education								
		Unadjusted^§^			Trend p-value^†^	Adjusted^§^			Trend p-value^†^
	Group	OR	95 % CI	p-value		OR	95 % CI	p-value	
Taking care of one’s diet for health^a^	Junior high/high school	1.00	(ref)		<0.001***	1.00	(ref)		<0.001***
	2-year college	1.67	1.35–2.06	<0.001***		1.37	1.10–1.71	0.006	
	4-year college/graduate school	1.66	1.37–2.00	<0.001***		1.68	1.37–2.05	<0.001***	
Eating vegetables^b^	Junior high/high school	1.00	(ref)		<0.001***	1.00	(ref)		<0.001***
	2-year college	1.48	1.22–1.80	<0.001***		1.23	1.00–1.50	0.049*	
	4-year college/graduate school	1.68	1.41–2.00	<0.001***		1.71	1.42–2.07	<0.001***	
Frequency of eating breakfast^c^	Junior high/high school	1.00	(ref)		0.954	1.00	(ref)		0.162
	2-year college	1.17	0.95–1.43	0.139		1.00	0.80–1.24	0.969	
	4-year college/graduate school	1.02	0.85–1.22	0.854		1.14	0.94–1.39	0.197	
Frequency of family breakfasts^c^	Junior high/high school	1.00	(ref)		0.650	1.00	(ref)		0.031*
	2-year college	1.22	0.99–1.50	0.066		1.01	0.81–1.27	0.932	
	4-year college/graduate school	1.07	0.87–1.30	0.470		1.25	1.01–1.54	0.040*	
Frequency of family dinners^c^	Junior high/high school	1.00	(ref)		<0.001***	1.00	(ref)		0.531
	2-year college	1.09	0.90–1.32	0.387		0.89	0.72–1.11	0.309	
	4-year college/graduate school	0.73	0.62–0.87	<0.001***		0.93	0.76–1.13	0.462	
Using the information on nutrition labels^b^	Junior high/high school	1.00	(ref)		<0.001***	1.00	(ref)		<0.001***
	2-year college	1.44	1.19–1.75	<0.001***		1.18	0.96–1.44	0.110	
	4-year college/graduate school	1.57	1.32–1.86	<0.001***		1.63	1.35–1.96	<0.001***	
Conversations with family or friends during meals^b^	Junior high/high school	1.00	(ref)		<0.001***	1.00	(ref)		<0.001***
	2-year college	1.52	1.25–1.85	<0.001***		1.20	0.98–1.48	0.078	
	4-year college/graduate school	1.60	1.34–1.91	<0.001***		1.70	1.40–2.06	<0.001***	

*P < 0.05; **P < 0.01; ***P < 0.001

^†^Trend test

^‡^The independent variable of education was adjusted for gender, age classification, marital status, residence status, employment status and household income

^§^OR = odds ratio; CI = confidence interval; ref = referent group

^||^As the dependent variable of dietary behaviors, seven items were confirmed the distribution of the answer and categorized in positive answer = 1, negative answer = 0

^a^Responses were given in six categories: (1) very often; (2) often; (3) sometimes; (4) rarely; (5) almost never; and (6) never. People who answered (1) to (3) to the question were defined as positive answer, (4) to (6) to the question were defined as negative answer

^b^Responses were in the following four categories: (1) very often; (2) often; (3) not much; and (4) never. People who answered (1) or (2) to the question were defined as positive answer, (3) or (4) to the question were defined as negative answer

^c^Responses were rated in the following five categories: (1) every day; (2) 4 or 5 days/week; (3) 2 or 3 days/week; (4) 1 day/week; and (5) never. People who answered (1) to the question were defined as positive answer, (2) or (5) to the question were defined as negative answer

Results of the logistic regression analysis of the association between household income and eating behaviors for men are shown in Table 5. In the unadjusted analysis, taking care of one’s diet for health, eating vegetables, frequency of eating breakfast, frequency of family breakfasts, using information on nutrition labels, and having conversations with family or friends during meals were positively associated with higher household income. After adjusting for all variables, frequency of family breakfasts and dinners were negatively associated with higher household income. The AOR of household income for frequency of family breakfasts and the 95 % CI calculated by residence status among men were: middle-income households with men living with other people: AOR 0.68, 95 % CI 0.48–0.97, *p* = 0.032; high-income households with men living with other people: AOR 0.70, 95 % CI 0.48—1.02, *p* = 0.065; and middle-income households with men living alone: AOR 0.82, 95 % CI 0.26–2.60, *p* = 0.730. It was impossible to estimate an AOR or 95 % CI for high-income households with men living alone because of the small sample size. Men with the highest levels of income were positively and significantly more likely than those with the lowest income levels to take care of their diet for the sake of their health, eat vegetables, use nutrition information, and converse during meals at the recommended levels. There was no significant association between frequency of eating breakfast and household income, but positive associations were seen between men’s household income, and the domains of taking care of one’s diet for health, eating vegetables, using nutrition information, conversations during meals, and negative association was seen between frequency of family breakfasts.

**Table 5:** Association of household income with eating behaviors in Japanese men: Unadjusted odds ratio (OR) and adjusted odds ratio (OR) and 95 % confidence interval (95 % CI)

Dependent variables^||^	Independent variables^‡^								
	Household income								
		Unadjusted^§^			Trend p-value^†^	Adjusted^§^			Trend p-value^†^
	Group	OR	95 % CI	p-value		OR	95 % CI	p-value	
Taking care of one’s diet for health^a^	<3,000,000 JPY	1.00	(ref)		<0.001***	1.00	(ref)		0.003**
	3,000,000 JPY–7,000,000 JPY	1.29	1.01–1.64	0.042*		1.18	0.90–1.55	0.234	
	>7,000,000 JPY	1.91	1.44–2.54	<0.001***		1.63	1.18–2.25	0.003**	
Eating vegetables^b^	<3,000,000 JPY	1.00	(ref)		<0.001***	1.00	(ref)		0.006**
	3,000,000 JPY–7,000,000 JPY	1.67	1.31–2.14	<0.001***		1.36	1.03–1.79	0.029*	
	>7,000,000 JPY	2.16	1.66–2.83	<0.001***		1.55	1.14–2.11	0.005**	
Frequency of eating breakfast^c^	<3,000,000 JPY	1.00	(ref)		0.098	1.00	(ref)		0.267
	3,000,000 JPY–7,000,000 JPY	1.26	0.99–1.60	0.060		0.96	0.74–1.26	0.783	
	>7,000,000 JPY	1.24	0.95–1.61	0.112		0.84	0.62–1.15	0.273	
Frequency of family breakfasts^c^	<3,000,000 JPY	1.00	(ref)		0.029*	1.00	(ref)		0.049*
	3,000,000 JPY–7,000,000 JPY	1.24	0.93–1.65	0.149		0.69	0.49–0.96	0.030*	
	>7,000,000 JPY	1.41	1.03–1.93	0.030*		0.67	0.47–0.98	0.037*	
Frequency of family dinners^c^	<3,000,000 JPY	1.00	(ref)		0.053	1.00	(ref)		<0.001***
	3,000,000 JPY–7,000,000 JPY	1.11	0.87–1.41	0.417		0.67	0.50–0.91	0.010*	
	>7,000,000 JPY	0.75	0.56–0.99	0.039*		0.38	0.27–0.54	<0.001***	
Using the information on nutrition labels^b^	<3,000,000 JPY	1.00	(ref)		<0.001***	1.00	(ref)		<0.001***
	3,000,000 JPY–7,000,000 JPY	1.29	1.00–1.65	0.047*		1.20	0.91–1.58	0.195	
	>7,000,000 JPY	2.14	1.64–2.80	<0.001***		1.89	1.39–2.57	<0.001***	
Conversations with family or friends during meals^b^	<3,000,000 JPY	1.00	(ref)		<0.001***	1.00	(ref)		0.018*
	3,000,000 JPY–7,000,000 JPY	1.34	1.03–1.73	0.029*		0.89	0.67–1.20	0.454	
	>7,000,000 JPY	2.43	1.85–3.20	<0.001***		1.38	1.01–1.91	0.044*	

*P < 0.05; **P < 0.01; ***P < 0.001

^†^Trend test

^‡^The independent variable of household income was adjusted for age classification, marital status, residence status, employment status and education

^§^OR = odds ratio; CI = confidence interval; ref = referent group

^||^As the dependent variable of dietary behaviors, seven items were confirmed the distribution of the answer and categorized in positive answer = 1, negative answer = 0

^a^Responses were given in six categories: (1) very often; (2) often; (3) sometimes; (4) rarely; (5) almost never; and (6) never. People who answered (1) to (3) to the question were defined as positive answer, (4) to (6) to the question were defined as negative answer

^b^Responses were in the following four categories: (1) very often; (2) often; (3) not much; and (4) never. People who answered (1) or (2) to the question were defined as positive answer, (3) or (4) to the question were defined as negative answer

^c^Responses were rated in the following five categories: (1) every day; (2) 4 or 5 days/week; (3) 2 or 3 days/week; (4) 1 day/week; and (5) never. People who answered (1) to the question were defined as positive answer, (2) or (5) to the question were defined as negative answer

In the unadjusted analysis for women (Table 6), most eating behaviors were positively associated with higher household income. After adjusting for all variables, frequency of family dinners was negatively associated with the highest household incomes. The AOR of household income for frequency of family dinners and the 95 % CI by residence status among women were: middle-income households with women living with others: AOR 0.83, 95 % CI 0.62–1.12, *p* = 0.225; high-income households with women living with others: AOR 0.65, 95 % CI 0.47–0.90, *p* = 0.010; and middle-income households with women living alone: AOR 1.90, 95 % CI 0.51–6.99, *p* = 0.337. The AOR and 95 % CI were impossible to estimate for high-income households with women living with others because of the small sample size. Women with the highest levels of income were positively and significantly more likely than those with the lowest levels of income to take care of their diet for the sake of their health, eat vegetables, use information on nutrition labels, and engage in conversation with family or friends during meals at the recommended levels. Frequency of eating breakfast and frequency of family breakfasts were not associated with household income. For household income among women, all domains showed significant trends.

**Table 6:** Association of household income with eating behaviors in Japanese women: Unadjusted odds ratio (OR) and adjusted odds ratio (OR) and 95 % confidence interval (95 % CI)

Dependent variables^||^	Independent variables^‡^								
	Household income								
		Unadjusted^§^			Trend p-value^†^	Adjusted^§^			Trend p-value^†^
	Group	OR	95 % CI	p-value		OR	95 % CI	p-value	
Taking care of one’s diet for health^a^	<3,000,000 JPY	1.00	(ref)		<0.001***	1.00	(ref)		<0.001***
	3,000,000 JPY–7,000,000 JPY	1.91	1.45–2.52	<0.001***		1.72	1.26–2.35	0.001**	
	>7,000,000 JPY	3.01	2.13–4.26	<0.001***		2.44	1.64–3.62	<0.001***	
Eating vegetables^b^	<3,000,000 JPY	1.00	(ref)		<0.001***	1.00	(ref)		<0.001***
	3,000,000 JPY–7,000,000 JPY	1.87	1.46–2.36	<0.001***		1.62	1.25–2.12	<0.001***	
	>7,000,000 JPY	2.54	1.95–3.33	<0.001***		1.99	1.46–2.71	<0.001***	
Frequency of eating breakfast^c^	<3,000,000 JPY	1.00	(ref)		0.004**	1.00	(ref)		0.088
	3,000,000 JPY–7,000,000 JPY	1.67	1.28–2.18	<0.001***		1.24	0.92–1.67	0.166	
	>7,000,000 JPY	1.50	1.12–2.02	0.007**		1.04	0.73–1.47	0.831	
Frequency of family breakfasts^c^	<3,000,000 JPY	1.00	(ref)		0.007**	1.00	(ref)		0.103
	3,000,000 JPY–7,000,000 JPY	1.68	1.31–2.16	<0.001***		1.02	0.77–1.36	0.895	
	>7,000,000 JPY	1.45	1.10–1.92	0.009**		0.78	0.57–1.08	0.135	
Frequency of family dinners^c^	<3,000,000 JPY	1.00	(ref)		0.004**	1.00	(ref)		0.010*
	3,000,000 JPY–7,000,000 JPY	1.71	1.35–2.17	<0.001***		0.86	0.64–1.15	0.299	
	>7,000,000 JPY	1.44	1.11–1.87	0.006**		0.66	0.48–0.92	0.014*	
Using the information on nutrition labels^b^	<3,000,000 JPY	1.00	(ref)		0.001**	1.00	(ref)		0.047*
	3,000,000 JPY–7,000,000 JPY	1.30	1.03–1.65	0.029*		1.28	0.98–1.66	0.071	
	>7,000,000 JPY	1.56	1.20–2.03	0.001**		1.37	1.01–1.86	0.044*	
Conversations with family or friends during meals^b^	<3,000,000 JPY	1.00	(ref)		<0.001***	1.00	(ref)		0.007**
	3,000,000 JPY–7,000,000 JPY	1.58	1.24–2.00	<0.001***		1.31	1.01–1.71	0.043*	
	>7,000,000 JPY	2.06	1.58–2.69	<0.001***		1.53	1.12–2.07	0.007**	

*P < 0.05; **P < 0.01; ***P < 0.001

^†^Trend test

^‡^The independent variable of household income was adjusted for age classification, marital status, residence status, employment status and education

^§^OR = odds ratio; CI = confidence interval; ref = referent group

^||^As the dependent variable of dietary behaviors, seven items were confirmed the distribution of the answer and categorized in positive answer = 1, negative answer = 0

^a^Responses were given in six categories: (1) very often; (2) often; (3) sometimes; (4) rarely; (5) almost never; and (6) never. People who answered (1) to (3) to the question were defined as positive answer, (4) to (6) to the question were defined as negative answer

^b^Responses were in the following four categories: (1) very often; (2) often; (3) not much; and (4) never. People who answered (1) or (2) to the question were defined as positive answer, (3) or (4) to the question were defined as negative answer

^c^Responses were rated in the following five categories: (1) every day; (2) 4 or 5 days/week; (3) 2 or 3 days/week; (4) 1 day/week; and (5) never. People who answered (1) to the question were defined as positive answer, (2) or (5) to the question were defined as negative answer

Results of the logistic regression analysis of the association between education and eating behaviors for men are shown in Table 7. In the unadjusted analysis, taking care of one’s diet for health, eating vegetables, using nutrition information, and conversing with family or friends during meals were positively associated with attainment of a 4-year college or graduate degree. Frequency of family dinners was negatively associated with completing 4-year college or graduate school. After adjusting for all variables, men who had graduated from 4-year college or graduate school were positively and significantly more likely than junior high school or high school graduates to take care of their diet for the sake of their health, eat vegetables, use the information on nutrition labels, and have positive conversations during meals at the recommended levels. Frequency of eating breakfast was not significantly associated with education. For education among men, the domains of taking care of one’s diet for health, eating vegetables, frequency of family dinners, using information on nutrition labels, and conversations during meals showed significant trends.

**Table 7:** Association of education with eating behaviors in Japanese men: Unadjusted odds ratio (OR) and adjusted odds ratio (OR) and 95 % confidence interval (95 % CI)

Dependent variables^||^	Independent variables^‡^								
	Education								
		Unadjusted^§^			Trend p-value^†^	Adjusted^§^			Trend p-value^†^
	Group	OR	95 % CI	p-value		OR	95 % CI	p-value	
Taking care of one’s diet for health^a^	Junior high/high school	1.00	(ref)		<0.001***	1.00	(ref)		0.001**
	2-year college	1.19	0.86–1.64	0.300		1.15	0.83–1.60	0.397	
	4-year college/graduate school	1.67	1.31–2.14	<0.001***		1.52	1.18–1.96	0.001**	
Eating vegetables^b^	Junior high/high school	1.00	(ref)		<0.001***	1.00	(ref)		<0.001***
	2-year college	1.29	0.93–1.79	0.123		1.27	0.91–1.78	0.160	
	4-year college/graduate school	1.74	1.36–2.22	<0.001***		1.65	1.27–2.14	<0.001***	
Frequency of eating breakfast^c^	Junior high/high school	1.00	(ref)		0.278	1.00	(ref)		0.247
	2-year college	0.90	0.66–1.24	0.528		0.93	0.67–1.28	0.656	
	4-year college/graduate school	1.12	0.88–1.42	0.372		1.14	0.88–1.47	0.318	
Frequency of family breakfasts^c^	Junior high/high school	1.00	(ref)		0.717	1.00	(ref)		0.633
	2-year college	0.92	0.63–1.34	0.654		0.92	0.62–1.38	0.690	
	4-year college/graduate school	1.04	0.78–1.37	0.798		1.06	0.78–1.44	0.714	
Frequency of family dinners^c^	Junior high/high school	1.00	(ref)		0.038*	1.00	(ref)		0.864
	2-year college	0.83	0.60–1.16	0.276		0.97	0.68–1.39	0.880	
	4-year college/graduate school	0.77	0.60–0.98	0.036*		0.98	0.74–1.29	0.873	
Using the information on nutrition labels^b^	Junior high/high school	1.00	(ref)		<0.001***	1.00	(ref)		0.008**
	2-year college	1.08	0.77–1.50	0.654		1.01	0.72–1.42	0.939	
	4-year college/graduate school	1.56	1.22–1.99	<0.001***		1.38	1.07–1.78	0.015*	
Conversations with family or friends during meals^b^	Junior high/high school	1.00	(ref)		<0.001***	1.00	(ref)		<0.001***
	2-year college	1.10	0.77–1.55	0.609		1.02	0.72–1.47	0.898	
	4-year college/graduate school	1.77	1.37–2.29	<0.001***		1.59	1.21–2.09	0.001**	

*P < 0.05; **P < 0.01; ***P < 0.001

^†^Trend test

^‡^The independent variable of education was adjusted for age classification, marital status, residence status, employment status and household income

^§^OR = odds ratio; CI = confidence interval; ref = referent group

^||^As the dependent variable of dietary behaviors, seven items were confirmed the distribution of the answer and categorized in positive answer = 1, negative answer = 0

^a^Responses were given in six categories: (1) very often; (2) often; (3) sometimes; (4) rarely; (5) almost never; and (6) never. People who answered (1) to (3) to the question were defined as positive answer, (4) to (6) to the question were defined as negative answer

^b^Responses were in the following four categories: (1) very often; (2) often; (3) not much; and (4) never. People who answered (1) or (2) to the question were defined as positive answer, (3) or (4) to the question were defined as negative answer

^c^Responses were rated in the following five categories: (1) every day; (2) 4 or 5 days/week; (3) 2 or 3 days/week; (4) 1 day/week; and (5) never. People who answered (1) to the question were defined as positive answer, (2) or (5) to the question were defined as negative answer

In the unadjusted analysis for women in Table 8, taking care of one’s diet for health, eating vegetables, using nutrition label information, and having conversations with family or friends while eating were positively associated with graduating from 4-year college or graduate school. After adjusting for all variables, women with 4-year college or graduate degrees were positively and significantly more likely than those with junior high or high school level education to take care of their diet for the sake of their health, eat vegetables, have frequent family breakfasts, use nutrition information, and converse during meals at the recommended levels. Frequency of eating breakfast was not associated with education. For education among women, the domains of taking care of one’s diet for health, eating vegetables, frequency of family breakfasts, using nutrition information, and positive conversations during meals showed significant trends.

**Table 8:** Association of education with eating behaviors in Japanese women: Unadjusted odds ratio (OR) and adjusted odds ratio (OR) and 95 % confidence interval (95 % CI)

Dependent variables^||^	Independent variables^‡^								
	Education								
	Group	Unadjusted^§^			Trend p-value^†^	Adjusted^§^			Trend p-value^†^
		OR	95 % CI	p-value		OR	95 % CI	p-value	
Taking care of one’s diet for health^a^	Junior high/high school	1.00	(ref)		<0.001***	1.00	(ref)		<0.001***
	2-year college	1.74	1.08–2.35	<0.001***		1.57	1.16–2.13	0.004**	
	4-year college/graduate school	2.08	1.52–2.85	<0.001***		1.93	1.39–2.70	<0.001***	
Eating vegetables^b^	Junior high/high school	1.00	(ref)		<0.001***	1.00	(ref)		<0.001***
	2-year college	1.36	1.06–1.75	0.015*		1.23	0.95–1.59	0.124	
	4-year college/graduate school	1.98	1.53–2.57	<0.001***		1.88	1.43–2.48	<0.001***	
Frequency of eating breakfast^c^	Junior high/high school	1.00	(ref)		0.678	1.00	(ref)		0.471
	2-year college	1.12	0.84–1.49	0.435		1.04	0.78–1.40	0.796	
	4-year college/graduate school	1.07	0.80–1.43	0.645		1.13	0.83–1.54	0.450	
Frequency of family breakfasts^c^	Junior high/high school	1.00	(ref)		0.041*	1.00	(ref)		0.020*
	2-year college	1.17	0.91–1.53	0.236		1.10	0.83–1.44	0.534	
	4-year college/graduate school	1.33	1.01–1.74	0.040*		1.42	1.05–1.90	0.021*	
Frequency of family dinners^c^	Junior high/high school	1.00	(ref)		0.197	1.00	(ref)		0.454
	2-year college	0.98	0.77–1.26	0.898		0.86	0.65–1.13	0.269	
	4-year college/graduate school	0.85	0.66–1.10	0.215		0.88	0.66–1.19	0.405	
Using the information on nutrition labels^b^	Junior high/high school	1.00	(ref)		<0.001***	1.00	(ref)		<0.001***
	2-year college	1.38	1.07–1.77	0.012*		1.33	1.04–1.72	0.026*	
	4-year college/graduate school	2.04	1.57–2.65	<0.001***		2.00	1.53–2.63	<0.001***	
Conversations with family or friends during meals^b^	Junior high/high school	1.00	((ref))		<0.001***	1.00	((ref))		<0.001***
	2-year college	1.43	1.11–1.84	0.005**		1.32	1.02–1.70	0.036*	
	4-year college/graduate school	1.85	1.42–2.39	<0.001***		1.81	1.37–2.38	<0.001***	

*P < 0.05; **P < 0.01; ***P < 0.001

^†^Trend test

^‡^The independent variable of education was adjusted for age classification, marital status, residence status, employment status and household income

^§^OR = odds ratio; CI = confidence interval; ref = referent group

^||^As the dependent variable of dietary behaviors, seven items were confirmed the distribution of the answer and categorized in positive answer = 1, negative answer = 0

^a^Responses were given in six categories: (1) very often; (2) often; (3) sometimes; (4) rarely; (5) almost never; and (6) never. People who answered (1) to (3) to the question were defined as positive answer, (4) to (6) to the question were defined as negative answer

^b^Responses were in the following four categories: (1) very often; (2) often; (3) not much; and (4) never. People who answered (1) or (2) to the question were defined as positive answer, (3) or (4) to the question were defined as negative answer

^c^Responses were rated in the following five categories: (1) every day; (2) 4 or 5 days/week; (3) 2 or 3 days/week; (4) 1 day/week; and (5) never. People who answered (1) to the question were defined as positive answer, (2) or (5) to the question were defined as negative answer

## Discussion

In this study using an Internet-based survey, eating vegetables, using the information on nutrition labels, and engaging in positive conversations with family or friends during meals were positively associated with higher household incomes and education levels among both men and women. Lower frequency of family breakfasts and dinners were associated with higher household income among men; lower frequency of family dinners was associated with higher household income among women. These associations were not seen with education. We found that eating behaviors differed by SES, suggesting that supporting healthy eating behaviors could reduce health disparities. To our knowledge, this is the first study to investigate the association of SES with healthy eating behavior in Japanese adults.

Our results showed that eating vegetables is a dietary behavior that is affected by SES. We set five small dishes of vegetables or about 350 g per day as a standard against which to assess frequency of vegetable intake. This is in line with a previous study in Japan [35], which suggested that the number of vegetable dishes consumed might be a simple and valid measure of vegetable intake and set an intake of 350 g per day as a standard. Those results predicted higher vegetable intake among those with higher SES and healthier eating habits, and may explain the association between household income and vegetable intake found in the NHNS [16]. In both Western countries and Japan, individuals with lower SES have a lower intake of healthy foods like vegetables [14, 16–18, 36, 37]. Many socioepidemiological studies in Japan have noted associations between SES and cancer risk [21] as well as cardiovascular disease and its risk factors [6, 36]. Increased vegetable intake is effective in preventing lifestyle-related cardiovascular disease and cancer [21]. At the behavioral level, we found that individuals with lower household incomes and education levels tended to eat vegetables less often. This highlights an urgent need for dietary intervention programs aimed at people with low SES, to promote vegetable-eating as a way to lower the risks of cancer and cardiovascular disease.

We found that participants with higher SES used the information on nutrition labels. Sinclair [28] reported that participants with higher household income who had attained medium to high education levels were significantly more likely to answer questions correctly on calorie intake than participants with lower education levels. In other reports, people who used the information on nutrition labels had healthier diets [27] and higher Healthy Eating Index scores [38]. We found that 46.3 % of Japanese adults used nutrition information, which is in line with results of the 2000 NHNS [39]. Our study results are the first of which we are aware to report an association between using information on nutrition labels and SES in Japan. A longitudinal study that includes SES factors is needed to examine the health effects of nutrition information use.

In our sample of Japanese adults, few with a household income of 7,000,000 JPY or more reported frequent family meals. This is a new implication in this area of study. Frequent family meals in adolescence and young adulthood may have a lasting positive influence on dietary quality and meal patterns, such as greater intake of green, yellow, and other vegetables and fruit [40]. Larson suggested that higher parental education level was positively associated with higher frequency of family meals among middle and high school students [24], and our findings are inconsistent with this. We found an initial positive association between household income and frequency of meals, but this became negative after adjustment. We added adjustment variables one by one and examined the results in detail, and found an effect attributable to residence status. A previous study found a high rate of skipping breakfast (15.1 %) [16] among men with the highest household income levels. In our study, we found a higher rate of skipping breakfast (37.2 %) in this group. We estimate that this is likely to be associated with a lower frequency of families eating breakfast together. Working women may also be unable to be at home for mealtimes because of the long working hours common in Japan (Table 5). About 25.6 % of employed men and women in Japan work are more than 49 h per week [41]. Japan is second only to South Korea among developed countries (high-income countries defined by the Organization for Economic Co-operation and Development) for its working time ratios [41, 42]. Kuroda reported that members of higher income households had longer working hours [43] than those with lower incomes. Woman may work even longer hours than men because of time spent doing housework [44]. The association between household income and the frequency of family meals did not change even by residence status, although we expected that having a family would lead to more talking at the dining table. For men with the highest household incomes living alone, the sample size was small and so could not be analyzed. We cannot therefore comment on the influence of residence status, the effect of adjusting for household income or relationships with the frequency of family meals. Further research with a larger sample size is necessary to address these issues. There are very few reports about the frequency of family meals among adults [45]. Although we think that household income and the frequency of family meals may be related to long working hours, we did not investigate these factors over the participants’ working lifetime. Further research is needed to determine whether this result is a specific social problem associated with the longer working hours seen in Japan.

Despite participants with higher household incomes showing lower frequency of family meals, these participants conversed more about food during mealtimes. A previous study showed that Japanese children who engaged in mealtime conversations during meals had better dietary attitudes, eating behaviors, and quality of life; good health status; higher vegetable intake; and good table manners [29, 30, 46]. The results of our study help to highlight the importance of positive conversation at the dining table, which may be influenced by working parents being unable to be at home for mealtimes.

This study had several limitations. First, our sample may not have been representative of the general population because we relied on an Internet-based survey. Caution should therefore be used in generalizing the results. Internet-based survey respondents may be more likely to have certain characteristics, such as being younger, having higher levels of education and income, and having better access to the Internet [47–49]. The research company used in this study, however, periodically analyzes and updates its registrant database. Second, there is room for improvement in the questionnaire. It was difficult to calculate individual-level equivalent income because participants self-reported their household income, so we had to use category levels. Good dietary practices have been shown to be associated with good health [50]. We did not, however, investigate certain confounding factors [14, 51] that are known to influence eating behaviors such as body mass index or health status, physical activity, and sleep status as control variables. We cannot therefore draw conclusions about the real impact of SES on eating behavior. Future research on SES and dietary habits is needed. Third, we chose to use seven items referenced in previous studies [20–30] and the NHNS [16] as healthy eating behaviors. There are, however, other possible healthy eating behaviors. Fourth, we cannot apply our results to people outside the target age ranges. For instance, future studies would be useful to compare our results to those for older adults and children or adolescents. Fifth, owing to the cross-sectional nature of the study, it is impossible to determine cause and effect between eating behaviors and household income or education. Despite these considerations, this study found statistically significant and positive associations between eating behaviors among Japanese adults, including eating vegetables, using the information on nutrition labels, conversing with family or friends during meals, and household income or education. It is important to examine the differences in eating behaviors among adults according to SES to facilitate early reductions in health disparity. A law was enacted in 2013 and poverty programs [31] were developed for children to reduce health disparities in Japan. The JAGES project [32] promoted the use of a large-scale socioepidemiological survey to prevent health disparities among older people in Japan. The associations between socioeconomic differences and eating behaviors in Japanese adults, however, have not been extensively investigated. It is important to promote the consumption of vegetables, as this has been shown to prevent the onset and progression of lifestyle-related diseases. Development of effective population-based program strategies to promote vegetable intake among people with low SES are also needed. Our next study will therefore aim to further identify the correlates [52] of eating behaviors by differences in SES.

## Conclusions

In this study, eating vegetables, using information found on nutrition labels, and engaging in positive conversations with family or friends during meals were significantly and positively associated with higher household income and education level among Japanese men and women. We identified a need to support eating behaviors that can help to reduce health disparities. SES should be considered in planning initiatives of health promotion and dietary improvement in Japan.

## Abbreviations

SES: socioeconomic status
WHO: World Health Organization
JAGES: Japan Gerontological Evaluation Study
OR: odds ratio
AOR: adjusted odds ratio
CI: confidence interval
NHNS: National Health and Nutrition Survey
